# Photochemical and Structural Studies on Cyclic Peptide Models

**DOI:** 10.3390/molecules23092196

**Published:** 2018-08-30

**Authors:** Tamás Milán Nagy, Krisztina Knapp, Eszter Illyés, István Timári, Gitta Schlosser, Gabriella Csík, Attila Borics, Zsuzsa Majer, Katalin E. Kövér

**Affiliations:** 1 Department of Inorganic and Analytical Chemistry, University of Debrecen, H-4032 Debrecen, Egyetem tér 1, Hungary; tamasmilan.nagy@science.unideb.hu (T.M.N.); timari.istvan@science.unideb.hu (I.T.); 2 Institute of Chemistry, Department of Organic Chemistry, ELTE Eötvös Loránd University, H-1518 Budapest, 112. P.O. Box 32, Hungary; knkriszta@gmail.com; 3 Chemie Ltd., H-1022 Budapest, Herman Ottó út 15, Hungary; eszter@vichem.hu; 4 Department of Analytical Chemistry, Institute of Chemistry, ELTE Eötvös Loránd University, H-1518 Budapest 112, P.O. Box 32, Hungary; schlosser@caesar.elte.hu; 5 Department of Biophysics and Radiation Biology, Semmelweis University Budapest, H-1428 Budapest, P.O. Box 2, Hungary; gabriella.csik@eok.sote.hu; 6 Institute of Biochemistry, Biological Research Centre, Hungarian Academy of Sciences, Temesvári krt. 62, H-6726 Szeged, Hungary

**Keywords:** cyclopeptides, UV irradiation, photolysis, disulfide bridges, NMR, MD

## Abstract

Ultra-violet (UV) irradiation has a significant impact on the structure and function of proteins that is supposed to be in relationship with the tryptophan-mediated photolysis of disulfide bonds. To investigate the correlation between the photoexcitation of Trp residues in polypeptides and the associated reduction of disulfide bridges, a series of small, cyclic oligopeptide models were analyzed in this work. Average distances between the aromatic side chains and the disulfide bridge were determined following molecular mechanics (MM) geometry optimizations. In this way, the possibility of cation–π interactions was also investigated. Molecular mechanics calculations revealed that the shortest distance between the side chain of the Trp residues and the disulfide bridge is approximately 5 Å in the cyclic pentapeptide models. Based on this, three tryptophan-containing cyclopeptide models were synthesized and analyzed by nuclear magnetic resonance (NMR) spectroscopy. Experimental data and detailed molecular dynamics (MD) simulations were in good agreement with MM geometry calculations. Selected model peptides were subjected to photolytic degradation to study the correlation of structural features and the photolytic cleavage of disulfide bonds in solution. Formation of free sulfhydryl groups upon illumination with near UV light was monitored by fluorescence spectroscopy after chemical derivatization with 7-diethylamino-3-(4-maleimidophenyl)-4-methylcoumarin (CPM) and mass spectrometry. Liquid cromatography-mass spectrometry (LC-MS) measurements indicated the presence of multiple photooxidation products (e.g., dimers, multimers and other oxidated products), suggesting that besides the photolysis of disulfide bonds secondary photolytic processes take place.

## 1. Introduction

Near UV-light-induced photochemical reactions have several applications in biotechnology and in medical sciences. Light-induced immobilization of proteins is a novel technology which can be utilized for covalent coupling of biomolecules on thiol-reactive surfaces in spatially oriented and/or spatially localized manner. Covalent immobilization using this emerging technology has been demonstrated for various proteins, such as for hydrolytic enzymes (lipases/esterases, lysozyme), proteases (human plasminogen), etc. [1,2]. The epidermal growth factor (EGF) receptor protein, often overexpressed in cancers and in other proliferative skin disorders, is rich in aromatic residues close to the disulfide bridges. It was demonstrated that the EGF receptor could be a promising target for laser-pulsed UV light treatment [3] to reduce the proliferative potential of cancer cells. Furthermore, UV light-induced immobilization could be also used for biomolecular screening techniques for diagnostic and therapeutic purposes [4].

Exposure to near-UV irradiation induces structural changes in proteins, that may alter their biological function [5,6]. One of the well-known processes of protein degradation is the near-UV light induced splitting of disulfide bonds which yields thiyl radicals (CysS) [7]. Thiyl radicals are formed via one-electron oxidation of cysteine residues [8] or through one-electron reduction of disulfide bonds [9,10]. UV irradiation-induced alteration of protein structures was extensively studied during the last decades. Photon absorption of peptide bonds, disulfide bridges, as well as side chains of aromatic amino acids (tryptophan, Trp; tyrosine, Tyr; phenylalanine, Phe) and histidine (His) was proposed to mediate the degradation process. Indole rings of Trp residues are the strongest near-UV absorbing groups in proteins, therefore, Trp residues are considered to be the primary components responsible for the photodegradation of proteins [2,5,6,7,11]. Light-induced, Trp-mediated breakage of disulfide bridges was investigated in detail on cutinase [11,12], bovine growth hormone [13], α-lactalbumin [6,14] and lysozyme [15] proteins. Determination of the required distances between aromatic amino acid side chains and disulfide bridges was the target of numerous studies, for example in the case of class I and class II major histocompatibility complex (MHC), mannose-binding lectin (MBL) subunit A, immunoglobulins, triosephosphateisomerase (TIM) barrel glycosidases, alkaline phosphatases and lysozymes [14,15,16]. Photodegradation of goat α-lactalbumin mutants containing substituted Trp residues revealed that the contribution of Trp residues to the photolytic breakage of the disulfide bonds depends strongly on the microenvironment of the Trp side chains and the disulfide bonds [6,14]. It was also demonstrated that thiyl radicals could be involved in several additional chemical reactions, such as recombination with other radical intermediates (Trp-, Tyr- and Phe radicals) [16], sulfenylation of the side chain of Trp (or Tyr/Phe), or recombination through interprotein cross-links, leading to dimerization [17,18]. Antibodies are also sensitive to photodegradation by near-UV light [19]. The formed thiyl radicals could generate both intra- [20] and interchain bonds which could lead to fragmentation and/or aggregation of these proteins [21].

Disulfide-bonded cystein residues with a neighboring Trp (Cys-Cys Trp structural triads) can be found in most of the protein families, but these are especially characteristic for immunoglobulins, lysozymes, and complement-associated proteins [22]. Lys and Tyr amino acids were also found to be preferred neighbors of this specific structural motif. Statistical analyses demonstrated that reduction-sensitive disulfide bonds are usually within the van der Waals distance (d ≤ 5Å) of photoactive aromatic groups (Trp, Tyr) [23,24]. However, the effect of neighboring aromatic groups could be exerted longer distances as well (d ~ 8–9 Å) [6,25,26].

Cation-π interactions, involving positively charged amino acid side chains (Lys, Arg) and an aromatic ring of Trp, Tyr or Phe might have a significant contribution to the stability of proteins [27] when the participating moieties are within 6.6 Å distance in a well-defined geometry [27,28,29]. According to statistical data, Arg residues are involved in these interactions in a larger extent than Lys residues and over one-fourth of Trp side chains participate in cation–π interactions [28]. These interactions could also have influence on peptide structures, but the stabilizing effect is usually much smaller [30].

To investigate the structural determinants of the aromatic group-mediated photolysis by near-UV light, a series of cyclic pentapeptides was designed and analyzed by molecular mechanics (MM) calculations. The peptide models contain a Trp residue as well as a disulfide bridge which limits conformational flexibility. Control peptides were also designed, in which Trp was replaced by Val (Table 1). Selected model peptides were also synthesized and illuminated by UV light at the absorbance maximum of Trp. The free sulfhydryl groups formed during the UV-illumination were detected through their CPM (7-diethylamino-3-(4-maleimidophenyl)-4-methylcoumarin) adducts, which have higher fluorescence absorbance than the CPM itself [31]. This paper reports on the MM calculations of the peptide models as well as the synthesis, photoreduction study and combined NMR and molecular dynamics (MD) based conformational analysis of the selected cyclic peptide models. In addition, LC-MS measurements were performed to estimate the amount of unreacted cyclopeptides of the studied models after the UV-illumination. LC-MS/MS experiments were also performed to identify the photolysis byproducts.

## 2. Results and Discussion

### 2.1. Design of Cyclic Peptide Models and Molecular Mechanics Calculations 

The size and flexibility of the disulfide-linked peptide macrocycle strongly influence the orientation of Trp side chains and so their distances from the respective S-atoms of the disulfide bridge. For the present photochemical and structural study, both symmetric and asymmetric cyclic pentapeptide models, containing a Trp residue and a disulfide bridge between the terminal Cys^1^ and Cys^5^ residues (Table 1), have been constructed and analyzed. Trp was replaced by Val in the corresponding reference compounds.

**Figure 1:**
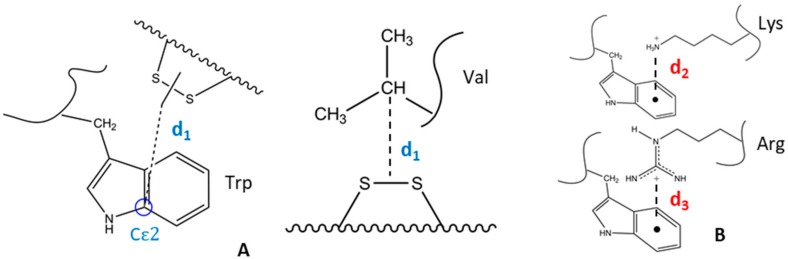
Scheme of calculated key distances: the distance (*d*_1_) between Trp/Val and the disulfide bridge (**A**); cation–π distance between the centroids of the benzene rings of the indole moieties of Trp and the cationic side chains of Lys (*d*_2_) and Arg(*d*_3_) (the ammonium nitrogen in Lys side chain or the guanidinium carbon in Arg side chain) (**B**).

MM calculations were performed to appoint optimal (ideal) peptide models, which fulfill structural criteria preferred in the photolytic processes. These structural parameters are the following: the distance between the Cε_2_ atom of Trp side chain and the center of disulfide bridge (*d*_1_) is 3–10 Å [22,24], the distance between the centroids of the benzene rings of the indole moieties of Trp and the cationic side chains of Lys (*d*_2_) and Arg residues (*d*_3_) are less than 6 Å [28,29]. Schematic representation of these key distances is shown on Figure 1. Corresponding structural characteristics of the designed peptides were determined by MM-based molecular modeling.

The results of molecular mechanics calculations indicated that the shortest distance (*d*_1_) between Trp and the SS bridge is approximately 5 Å in the designed cyclic pentapeptide models (Table S1). The Trp side chain and the disulfide bridge were found in the most favorable positions for interaction in Ac-c(CAWAC)-NH_2_, Ac-c(CWAKC)-NH_2_ and Ac-c(CWKAC)-NH_2_ peptides, in which the relevant distances (*d*_1_) were between 5.5–7.9 Å. In contrast to that, in Ac-c(CWAGC)-NH_2_ this distance was found to be *d*_1_ = 9.6 Å (Table S1). Based on MM calculations, the Arg-containing models (Table S1) do not fulfill the essential distance criteria of cation–π interactions. The Lys-containing peptide models, however, match such requirements. Based on the results of these calculations, the following cyclic pentapeptides were identified as possible targets of photoactivated disulfide reduction: Ac-c(CWAGC)-NH_2_ < Ac-c(CWKAC)-NH_2_ < Ac-c(CAWAC)-NH_2_, where the order indicates the degree of predicted photosensitivity. These three peptides (Table 1) were then selected and synthesized for further structural studies and photodegradation experiments. The corresponding linear peptides were made by solid phase peptide synthesis (SPPS) [32], and these precursors were cyclized afterwards in solution [32,33]. Analytical data (MW) of the peptides and calculated distances are summarized in Table 1.

### 2.2. Determination of Key Distances by NMR and MD Analyses

Distances relevant for photolytic activity were analyzed by combined NMR and MD methods, through exhaustive conformational analysis of the three selected Trp-containing pentapeptide models: Ac-c(CWAGC)-NH_2_, Ac-c(CWKAC)-NH_2_ and Ac-c(CAWAC)-NH_2_. To explore the role of the amino group close to the Trp residue and to study the cation–π interactions, peptides with and without Lys residues were also examined.

#### 2.2.1. NMR Analysis

Sequential (i ± 1), medium range (i ± 2,3) rotating-frame Overhauser effects (ROEs) and the contacts of the Trp residue were assigned in the rotating-frame Overhauser effect spectroscopy (ROESY) spectra in order to determine the pertinent conformational features of the peptides and to assess the spatial proximity of the indole ring to the disulfide bridge (Trp-SS).

**Figure 2:**
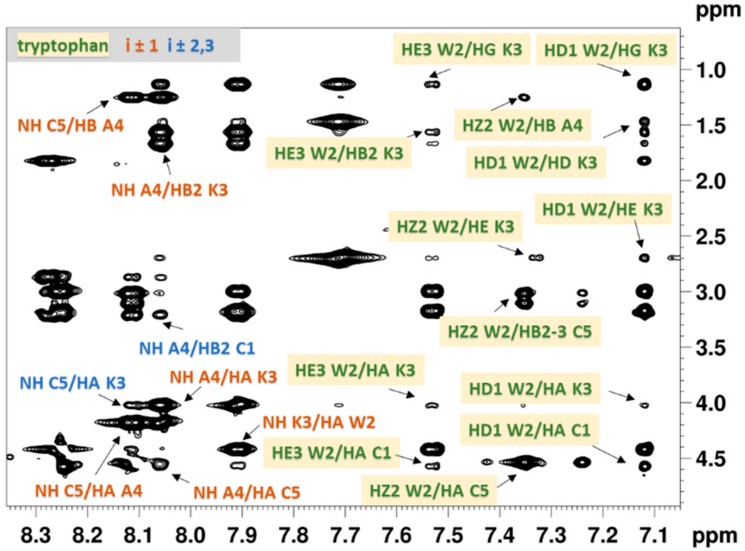
Expanded region of the ROESY spectrum of Ac-c(CWKAC)-NH_2_ measured in DMSO-*d*_6_ at 298 K with the mixing time of 150 ms. Sequential (i ± 1) and medium range (i ± 2,3) ROE peaks are labelled by orangeand blue, respectively. Interresidual ROEs of Trp are highlighted by yellow boxes.

Besides the sequential and medium range ROEs observed for Ac-c(CWKAC)-NH_2_, the ROESY spectrum (Figure 2) shows cross peaks between Trp and Cys residues with significant intensity (e.g., HZ2 Trp^2^/HB2 Cys^5^) suggesting the spatial proximity of the Trp ring to the disulfide bridge. In addition, almost all of the Trp ring H-atoms show ROE cross peaks with the side chain of Lys, indicating the presence of cation–π interaction. The ROE patterns observed in the ROESY spectra of Ac-c(CAWAC)-NH_2_ and Ac-c(CWAGC)-NH_2_ (Figure S1) also support the Trp-SS proximity. 

**Figure 3:**
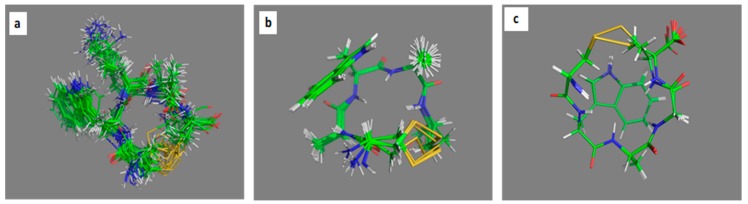
ROE-based structural ensembles. (**a**): Ac-c(CWKAC)-NH_2_, (**b**): Ac-c(CAWAC)-NH_2_ and (**c**): Ac-c(CWAGC)-NH_2_. The starting structures for MD refinement were extracted from these NMR ensembles as described in the experimental and the supplementary section (Table S2).

Besides qualitative analyses of ROESY spectra the ROE-derived proton–proton distances were utilized as restraints in the subsequent iterative structure refinement, yielding conformational ensembles shown in Figure 3. 

#### 2.2.2. MD Analysis of Key Interatomic Distances

**Figure 4:**
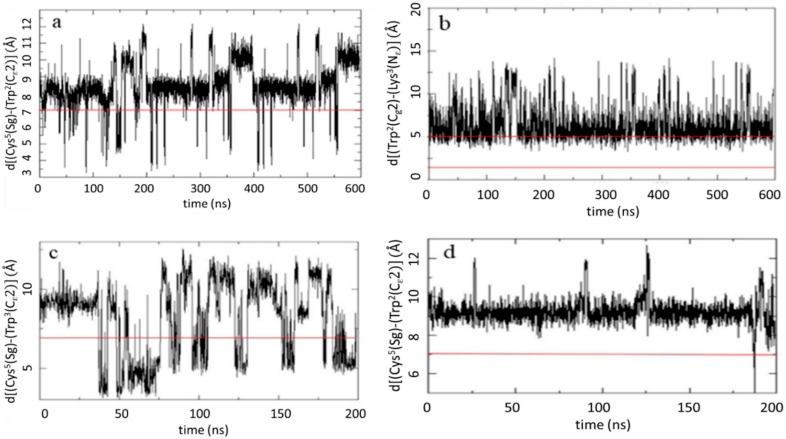
Key interatomic distances (in Å) relevant for photolytic processes. The required criteria are represented by red lines. Distance between the disulfide bridge and the Trp ring d[Cys^5^(S_γ_)-Trp^2–3^(C_ε_2)] along three concatenated (**a**: Ac-c(CWKAC)-NH_2_) and 200 ns long (**c**: Ac-c(CAWAC)-NH_2_, **d**: Ac-c(CWAGC)-NH_2_) MD-trajectories. Distance between Trp ring and Lys side chain d[Trp^2^(C_ε_2)-K^3^(N_ε_)] along three concatenated MD-trajectories (**b**: Ac-c(CWKAC)-NH_2_).

Analyzing the MD-trajectory in Ac-c(CWKAC)-NH_2_ (Figure 4a), the distance (*d*_1_) between C_ε_2 atom of Trp side chain and the center of disulfide bridge is found to be mostly above 7 Å with an average of 8.55 ± 1.34 Å. The population of conformers having favorable distance (<7 Å) of the aromatic and disulfide groups was found to be around 7% for this peptide. The close contact of the respective residues is supported by the observed ROEs as well (Figure 2). In contrast, for Ac-c(CAWAC)-NH_2_, the most flexible peptide, a significant population (33%) of conformers with distance *d*_1_ below 7 Å was found, giving an average of 8.12 ± 2.40 Å (Figure 4c). In Ac-c(CWAGC)-NH_2_, the least flexible peptide, the indole ring did not show up in the proximity of the disulfide bridge. The corresponding disulfide-Trp C_ε_2 distance *d*_1_ was 9.21 ± 0.62 Å along the 200 ns MD trajectory (Figure 4d). 

The only peptide with a protonated Lys residue, in which the contribution of possible cation–π interaction could be examined, is Ac-c(CWKAC)-NH_2_. MD results demonstrated that the flexible side chain of Lys^3^ is likely to be positioned near the aromatic ring of Trp^2^, which is in agreement with the ROE contacts shown in Figure 2. The corresponding average distance is 6.38 Å with a deviation of ±1.96 Å. In 18% population of conformers this distance fell in the range of 2 Å < *d*_2_ < 5 Å (Figure 4b). The distance between groups being putatively involved in photolysis through the formation of a super-oxide radical of the Trp carbonyl oxygen (CO) was also investigated for each peptide. In Ac-c(CWKAC)-NH_2_, Trp^2^-CO was located close to the disulfide bridge, at an average distance of 3.62 ± 1.01 Å. In Ac-c(CAWAC)-NH_2_, the corresponding average distance was 5.31 ± 1.44 Å, while in the most rigid peptide, Ac-c(CWAGC)-NH_2_, a relatively long distance (8.16 ± 0.75 Å) was observed.

### 2.3. Comparative MD Analysis of Conformational Ensembles in Dimethyl Sulfoxide and Water

Although the NMR measurements could be carried out only in dimethyl sulfoxide (DMSO-*d*_6_) due to low water solubility of the studied peptides, but to disclose the potential impact of solvent on the conformational ensembles, MD calculations were performed in both DMSO and water.

**Figure 5:**
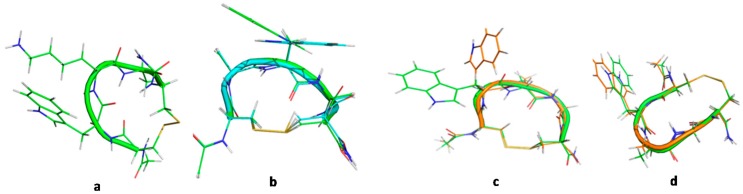
Representative models (geometry optimized middle structures) of the studied peptides obtained from clustering of the MD ensembles. (**a**): Ac-c(CWKAC)-NH_2_ in H_2_O, cluster 1 type II β-turn (green tube), *d*_1_(Trp-SS): 9.82 Å, *d*_2_(Trp-Lys): 5.20 Å; (**b**): Ac-c(CAWAC)-NH_2_ in H_2_O, clusters 1 and 2, type IV β-turn (green and blue tubes), *d*_1_(Trp-SS): 9.44 and 8.83 Å, respectively; (**c**): Ac-c(CWAGC)-NH_2_ in DMSO, clusters 1 and 4, type IV β-turn (green and orange tubes), *d*_1_(Trp-SS): 9.1 and 8.6 Å, respectively; (**d**): Ac-c(CWAGC)-NH_2_ in DMSO, clusters 3 and 5, type II β-turn (green and orange tubes), *d*_1_(Trp-SS): 10.25 and 10.19 Å, respectively.

The structure of Ac-c(CWKAC)-NH_2_ was found to be similar in both water and DMSO. This is supported by the secondary structure analysis of the MD-derived structural ensembles reported in Table 2. The largest conformational families adopt unordered backbone structures, while the other conformational groups adopt type II- (Figure 5a) or type IV-β-turns. Comparing their representative structures, significant differences can be observed in the side-chain orientation of Trp. 

Cluster analysis of the MD ensembles of Ac-c(CAWAC)-NH_2_ with regard to backbone and side chain conformations indicated that both backbone and the Trp^3^ side chain possess enhanced flexibility and the peptide adopts mainly unordered conformations. Minor conformational families of type-IV β-turn with different orientations of the Trp ring exist in both solvents (Figure 5b), but with considerably reduced population in DMSO. 

In the case of Ac-c(CWAGC)-NH_2_, a large conformational family, stabilized by a type-I β-turn was observed in water besides the unordered backbone conformations. In contrast, a type-II β-turn structural family (Figure 5d) and a type-IV β-turn family (Figure 5c) were found in DMSO, where this latter family can be further subdivided on the basis of Trp^2^ side chain conformation: one adopted *gauche*+ rotameric state (χ_1_ = 67°), while the other preferred *trans* (χ_1_ = 179°). In comparison with the other peptides, the populations of the unordered structures in Ac-c(CWAGC)-NH_2_ were lower in both solvents.

In summary, comparing the results of MD simulations performed in the two solvents (H_2_O, DMSO), it can be concluded that the conformational equilibria of Ac-c(CWKAC)-NH_2_ and Ac-c(CAWAC)-NH_2_ do not show significant differences, neither in the type nor in the population of the adopted secondary structures of peptide backbone. On the other hand, in the case of the most rigid Ac-c(CWAGC)-NH_2_, significant effect of the solvent was observed. Namely, type I β-turn structures were favored in water, and type II β-turns in DMSO.

### 2.4. Analysis of Hydrogen Bonds

NMR data and MD trajectories were also examined for structure-stabilizing hydrogen bonds. The small temperature dependence of amide proton chemical shifts predicts exchange-protected, H-bonded amide protons of the third residues in Ac-c(CWKAC)-NH_2_ (Lys^3^) and Ac-c(CAWAC)-NH_2_ (Trp^3^) with −1.7 and −1.6 ppb/K temperature gradients (Table 3), respectively. This may indicate a population of β-turn structures, stabilized through a H-bond between Lys^3^/Trp^3^(NH) and Cys^1^(CO). In contrast, all amide protons of Ac-c(CWAGC)-NH_2_ show large temperature effect (ranging from −4.8 to −5.8 ppb/K), thus no H-bonded sites could be confirmed by NMR.

Geometrical restraint calculations for hydrogen bonds in the structures of MD ensembles were carried out in parallel, searching for donor-acceptor pairs (Table S3). In DMSO, the populations of conformers having possible H-bonds are reduced in comparison to water. However, the same pattern (donor-acceptor pairs) was observed in both solvents. The occurrence of stabilizing H-bonds between Cys^1^ CO and residue 3 NH in all three peptides was confirmed. In Ac-c(CWKAC)-NH_2_ and Ac-c(CAWAC)-NH_2_ H-bonds between Trp^3^ NH and Cys^5^ CO were also established. In addition, the contribution of protecting groups to structure stabilization was also observed. The acetyl group CO likely accepts amide-NHs from the proximal residues as well as the *C*-terminal amide NH, which is a good donor for Cys^5^ CO as well. 

In conclusion, the presence of H-bond involving NH of residue 3 in Ac-c(CWKAC)-NH_2_ and Ac-c(CAWAC)-NH_2_ has been corroborated by both experimental NMR data and MD statistics. In the case of Ac-c(CWAGC)-NH_2_, differences were observed between the H-bond patterns predicted with the two approaches, which could be likely explained by possible conformational exchange processes resulting in enhanced temperature gradients.

### 2.5. NMR and MD Analysis of Trp Side-Chain Conformation

The conformational flexibility of the Trp side chain being critical with regard to the photolytic mechanism was assessed by NMR. The population of the three staggered rotamers (*gauche*(+), *gauche*(−), *trans*) was assessed from the relevant vicinal proton–proton coupling constants (^3^*J*_Hβ,Hβ′-Hα_) measured in pure absorption phase 2D TOCSY (TOtal Correlation SpectroscopY) spectra (Ac-c(CWKAC)-NH_2_: ^3^*J*_Hβ-Hα_ = 6.1 Hz, ^3^*J*_Hβ’-Hα_ = 8.0 Hz and Ac-c(CWAGC)-NH_2_: ^3^*J*_Hβ-Hα_ = 4.8 Hz, ^3^*J*_Hβ’-Hα_ = 9.6 Hz), utilizing the Pachler-equation. The stereospecific assignment of β-protons was deduced from the intensity pattern of NH/H_β_ and H_α_/H_β_ ROESY cross peaks (Figure S1). In Ac-c(CAWAC)-NH_2_, the geminal β-proton resonances could not be resolved due to likely conformational exchange averaging. The NMR-derived rotamer populations summarized in Table 4 show that in DMSO solutions all three conformational states are accessible to both Ac-c(CWKAC)-NH_2_ and Ac-c(CWAGC)-NH_2_ with a preference for the *trans* rotamer in Ac-c(CWKAC)-NH_2_ and for the *gauche*(−) in Ac-c(CWAGC)-NH_2_, respectively.

According to the MD ensembles, the distributions of the χ^1^ torsion angle in Ac-c(CWKAC)-NH_2_ and Ac-c(CAWAC)-NH_2_ show nearly the same pattern in both solvents. In contrast, a significant solvent effect was observed in Ac-c(CWAGC)-NH_2_. The most dominant rotamer with 93% propensity was *gauche* (+) in water, while in DMSO the rotamer distribution became more evenly balanced with contributions from all three rotamers but with definite preference toward the *gauche*(−) state. It should be noted that rotamer populations being available from both the NMR and MD analyses (Table 4) are generally in excellent agreement, providing further proof of the proposed structural features.

In conclusion, based on the detailed MD calculations being supported by experimental NMR data, our results indicate that the structural characteristics of the three selected Trp-containing peptides conform to the predicted features, suggested on the basis of molecular mechanics force field calculations. This means that the peptides synthesized and investigated in the present work are suitable models for studying the structural aspects and determinants of the photolytic cleavage of disulfide bonds and thereby to prove or disprove the different hypotheses proposed in the literature so far.

### 2.6. Quenching Effect of Disulfide Bridges in Peptide Models

**Figure 6:**
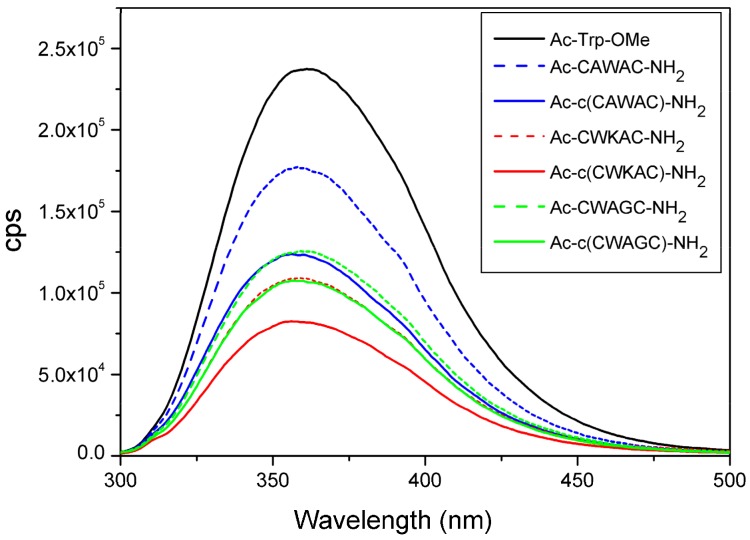
Trp-fluorescence measurements of linear and cyclic peptide models. Emission spectra were measured at λ_ex_ = 280 nm.

To study the effect of disulfide bridges on fluorescence emission, spectra of linear and cyclic model peptides at λ_ex_ = 280 nm were measured under similar conditions (Figure 6). Ac-Trp-OMe derivative was used as reference compound to mimic the peptide-bound Trp residue. The intensities/amplitudes of spectra vary according to the amino acid composition and sequence of the peptides, and according to their linear or cyclic nature. 

The emission intensities of cyclic peptides are systematically lower compared to the Ac-Trp-OMe derivative or to the linear peptide variants with similar amino acid compositions. This phenomenon can be explained by the quenching effect of disulfide bridges present in cyclic peptides [34,35,36]. The difference in fluorescence intensities of cyclic peptide models can be explained by the different distances (*d*_1_) between Trp residues and SS bonds (i.e., the quenching effect of the SS bond). We propose that the difference in Trp-fluorescence intensity could provide qualitative information about the spatial proximity of the SS bond and the Trp-chromophore (Figure 6 and Table S4) or about a different orientation of the Trp side chain. However, quenching effect of the peptide backbone as well as of the disulfide bond could also modify the data. Linear peptide models and the reference compound Ac-Trp-OMe show higher fluorescence intensity possibly due to the lack of disulfide bridge and the conformational diversity of linear peptides. 

### 2.7. UV Irradiation of Peptides Monitored by Measuring the Amount of Evolving Thiol Groups with Fluorescence Spectroscopy

Changes in the fluorescence spectra were registered as a function of the absorbed light energy after UV irradiation together with the determination of the amount of free thiol groups formed. To distinguish between degradation processes induced by light absorption of Trp and other less specific light-induced reactions, control measurements were performed using Val-substituted disulfide bridged cyclic pentapeptides lacking the aromatic Trp residue. 

**Figure 7:**
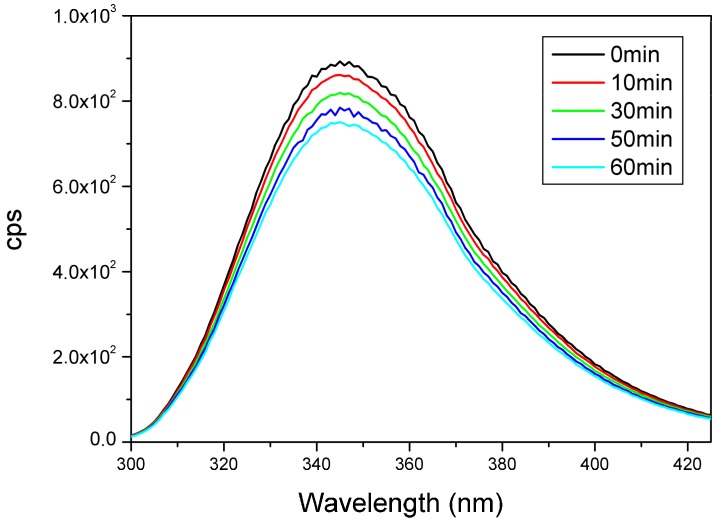
Trp fluorescence spectrum of peptide Ac-c(CWAGC)-NH_2_ after irradiation at 280 nm ―: 0 min, ―: 10 min, ―: 30 min, ―: 50 min, ―: 60 min.

Peptides were irradiated in solution with UV light at 280 nm (see Experimental). The fluorescence emission spectra of the cyclic peptides irradiated with various time periods are shown in Figure 7 and Figure S2. Significant (15–20%) decrease in Trp fluorescence intensities was observed with increasing duration of irradiation time in the case of all Trp-containing model peptides. Extended irradiation time (2–3 h) resulted in a significant, about 30% decrease in the Trp fluorescence intensities (Figure 7 and Figure S3A). Note that similar results were obtained for goat α-lactalbumin [6,14]. 

The decrease of the fluorescence intensity shown on Ac-c(CWAGC)-NH_2_ (Figure 7) can be explained by the photodestruction of the fluorophore, therefore 1-h illumination time was chosen for further studies.

**Figure 8:**
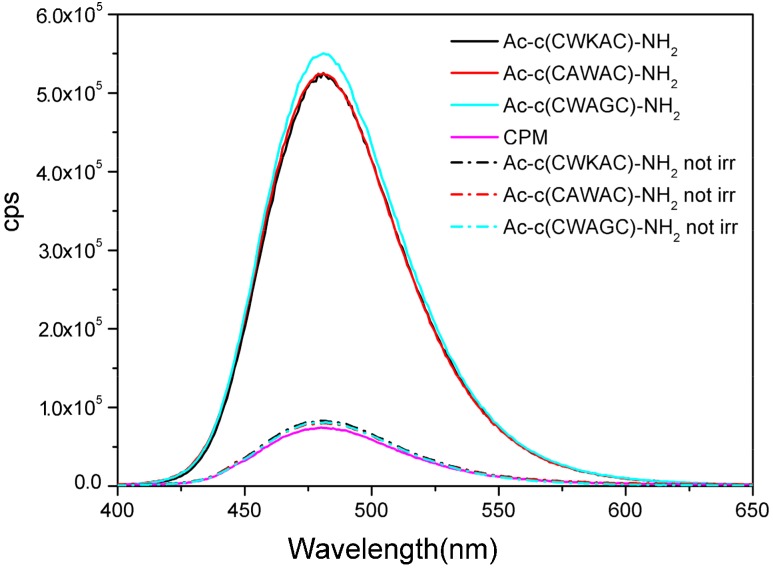
Detection of free sulfhydryls by fluorescence emission spectroscopy: CPM–peptide adducts after 1h illumination of the peptides at 280 nm (λ_ex_ = 387 nm). CPM was added to the not illuminated samples for control (dash lines).

Formation of free sulfhydryl groups under UV irradiation of peptides was detected by chemical derivatization with CPM reagent. CPM reacts with free sulfhydryl groups immediately and the reaction is complete within 10 min. The formed CPM-peptide adducts are stable for a long time. Due to the high fluorescence intensity of the adducts, the reaction is suitable for the sensitive detection of sulfhydryl formation. In the experiments, a model peptide was designed and synthesized as calibration standard. This linear peptide, Ac-CWAKC(Acm)-NH_2_, contained one free sulfhydryl group, while the second sulfhydryl group was blocked by an acetamidomethyl (Acm)-protecting group [37] (Figure S4). The impact of a protonated amino group near to Trp (cation–π interaction) was also investigated using peptides with and without Lys in the sequence. Figure 8 shows the fluorescence intensity of the CPM adducts of the selected peptides after 1 h of illumination. 

Interestingly, under these conditions, no significant difference was observed between the peptides after UV irradiation, as presented by the data in Table 5.

The data (Table 5) verified that the photodegradation of the SS bridge occurs in the studied peptides, and the quantity of the free sulfhydryl groups evolved is between 5–6% in all case. The low amounts of detected sulfhydryl groups can be explained by their reactivity and also by a fast conversion to other products (see paragraph 2.8 below). However, fluorescence measurements do not support the structure-based predictions of the photodegradation efficacy. It is important to note that the control model peptide containing Val instead of Trp also produced free thiols in detectable amount. 

Moziccionacci [38] and coworkers reported similar results earlier after illuminating nonaromatic Cys-containing hexapeptides. The UV-irradiation of a peptide disulfide bond generates thiyl radicals which form two main products, namely the cysteine and the thioaldehyde-containing peptides. This proton transfer occurs prior to the formation of final, nonradical products including free thiol, thioaldehyde, and aldehyde. Thiyl radicals in peptides and proteins may also be involved in reversible, intramolecular proton abstraction reactions from nearby Cα-H or side chain C–H bonds generating intermediary C-centered radicals. Their results demonstrate that the thiyl radicals of Cys react with the Val residues but also enter a pathway which yields a new reaction product, tentatively identified as isothiazol-3(2H)-one [39]. We suppose that these alternative photodegradation pathways also influence the formation of free sulfhydryl groups from the cyclic peptide models.

### 2.8. LC-MS Measurements

To investigate the possible secondary photolytic processes originating from radicals in the peptide solutions after UV-irradiation, mass spectrometric experiments were performed. The amount of unreacted cyclic peptides after the illumination was estimated by LC-MS measurements. To have a qualitative overview of the degradation of the peptides, three parallel experiments were performed for both the illuminated and non-illuminated peptides. The percentage of the unreacted peptides was calculated from the integrated mass peak intensities. The results verified the significant decrease in the amount of the cyclic model peptides. In the case of peptide Ac-c(CWAGC)-NH_2_, approximately 80% of the peptide remained intact after 1 h illumination time. Other peptides exhibited even lower survival: approx. 65% of peptide Ac-c(CAWAC)-NH_2_ remained intact, while Ac-c(CVAKC)-NH_2_ and Ac-c(CWKAC)-NH_2_, showed the lowest values with approx. 45% survival only. Our data therefore confirm the occurrence and importance of secondary photolytic processes, leading to alternative byproducts beside the formation of free sulfhydryls.

**Figure 9:**
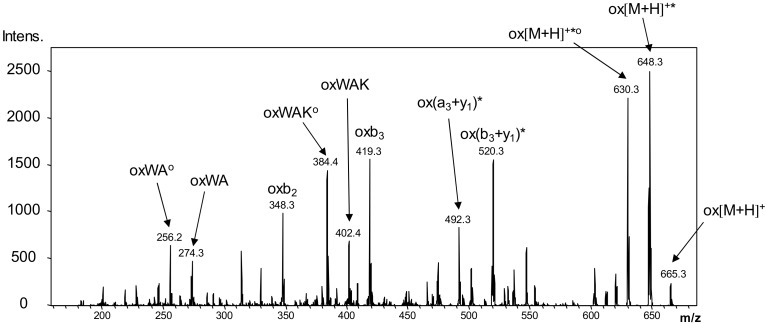
LC-MS/MS fragmentation profile of the oxidized Ac-c(CWAKC)-NH_2_ peptide, confirming the location of the site of oxidation on the Trp residue. The spectrum was acquired on a Bruker Esquire 3000+ ion trap tandem mass spectrometer. * is used to label the neutral loss of ammonia, ^o^ is used for water loss. Fragment ions in brackets are connected through a disulfide bridge.

Beside the formation of the CPM adducts of the peptides, LC-MS experiments confirmed the presence of oxidized products, such as the oxidation of the Trp residue (Figure 9) as well. In addition to oxidation, a major side reaction was the dimerization of the peptides through newly formed disulfide bridges. This can be explained with the fast rearrangement of the disulfide bridges under pH 7.5 upon illumination, resulting in the formation of head–tail- or head–head-oriented peptide dimers. Our results show that the indolyl side chain of Trp is highly sensitive in the near UV region, therefore, UV irradiation results in the formation of complex mixtures, even in the case of the studied simple model peptides.

## 3. Experimental

### 3.1. Materials

Diisopropylcarbodiimide (DIPCI), 1-hydroxybenztriazole (HOBt), and trifluoroacetic acid (TFA) were obtained from Fluka. Acetonitrile (MeCN), dichloromethane (DCM), and dimethylformamide (DMF) are from Reanal (Budapest, Hungary). The amino acids were represented by three- and one letter code. The protected amino acids (Fmoc-Gly-OH, Fmoc-Ala-OH, Fmoc-Cys(Acm)-OH, Fmoc-Lys(Boc)-OH, Fmoc-Trp(Boc)-OH, Fmoc-Tyr(tBu)-OH, Fmoc-Cys(Trt)-OH, were purchased from Reanal (Budapest, Hungary). (Acm- acetamidomethyl, Fmoc—9-fluorenylmethoxycarbonyl, Trt—trityl, Boc—tert-buthyloxycarbonyl, tBu—tert-butyl). Rink-Amid MBHA resin was from Novabiochem (Darmstadt, Germany). 7-diethylamino-3-(4′-maleimidophenyl)-4-methylcoumarine (CPM) from Molecular Probes (Eugene, Oregon, OR, USA), TIS—triisopropylsilane from Sigma Aldrich (Saint Louis, MO, USA). 

### 3.2. High-Performance Liquid Cromatography (HPLC)

Analytical RP-HPLC was carried out on a Jasco-HPLC (Jasco-2000 analytical system, Tokyo, Japan) instrument using a Phenomenex Gemini-NX (Torrance, CA, USA) (5 µm, 4.6 mm × 150 mm, 110 Å) column.0.08% (*v/v*) TFA in water (A) and 0.06% (*v/v*) TFA in acetonitrile (B) were used as eluents. Analytical gradient was: 5–50% B in 25 min. Flow rate was 0.7 mL/ min. Purification of the peptides was performed by preparative HPLC by Knauer instrumentation using Phenomenex Gemini-NX (5 μm, 21.2 mm × 150 mm, 110 Å) column, with 10 mL/min flow rate.

### 3.3. LC-MS

Analytical LC-MS measurements were performed on a Waters Acquity SQD MS system equipped with Alliance 2795 separation module using reverse phase column (Herts, UK). 

UV spectra were recorded using a Waters 996 DAD UV detector. Method: Waters XBridge C18 column (5 cm × 4.6 m, 3.5 µm), gradient elution 0–95% B over 7.00 min. A eluent: MilliQ water containing 0.1% formic acid, B eluent: LC-MS grade acetonitrile. 50 µL samples were injected from 35-μM illuminated and not-illuminated peptide solutions.

### 3.4. LC-MS/MS

LC-MS/MS tandem mass spectrometric experiments were performed on a Bruker Esquire 3000+ ion trap mass spectrometer (Bremen, Germany) equipped with electrospray ionization (ESI) source.

### 3.5. MM Calculation

MM calculations were performed with HyperChem 6.0 (Hypercube, Inc., Gainesville, FL, USA) using Amber 96 force field in vacuum (Newton–Raphson and Polak–Ribiere algorithms, 2000 cycles). Optimization was started from structures obtained by setting trans amide bonds and SS distance to 2.04 Å. During the conformational search φi, ψi, ξi and χi torsion angles were scanned.

### 3.6. NMR Spectroscopy and Structure Calculation

NMR measurements have been carried out at 298 K using a 500 MHz Bruker Avance II spectrometer (Bruker BioSpin GmbH, Rheinstetten, Germany) equipped with TXI (^1^H/^13^C/^15^N triple resonance) and BBI (multinuclear), z-axis gradient, 5-mm probes 9.3 mg, 11.4 mg, and 8.0 mg of peptide samples (Ac-c(CAWAC)-NH_2_, Ac-c(CWAGC)-NH_2_ and Ac-c(CWKAC)-NH_2_ respectively) were dissolved in 0.5 mL of DMSO-*d*_6_. Chemical shifts were referenced to the residual solvent signal (^1^H, DMSO-*d*_6_, δ = 2.49 ppm, and ^13^C, DMSO-*d*_6_ δ = 39.5 ppm). All of the spectral data were extracted from a series of 1D and 2D spectra, including ^1^H-^1^H COSY (COrrelation SpectroscopY), ^1^H-^1^H TOCSY, ^1^H-^1^H ROESY and ^1^H-^13^C HSQC (Heteronuclear Single Quantum Correlation) recorded for each peptide.

The gradient enhanced, zero-quantum filtered, pure absorption phase ^1^H-^1^H TOCSY spectra were recorded using MLEV-17 sequence [40,41] with a mixing time of 60 ms. The 2D data matrix consisted of 4096 and 512 total data points. A spin-lock field of 8300 Hz was used for the TOCSY transfer. A relaxation delay of 1.7 s was allowed between scans. Zero-filling in F1 and a squared cosine function in both F1 and F2 were applied prior to Fourier-transformation.

The ^1^H-^1^H ROESY spectra were recorded using the conventional ROESY experiment [42,43] with a spin-lock field of 3300 Hz and 150 ms duration of mixing. The number of scans was 32 in each experiment and the 2D data matrix consisted of 2048 and 512 total data points. These matrices were zero filled and apodized by a squared cosine function in both dimensions.

The ^1^H-^13^C HSQC correlation maps were recorded using the standard Bruker pulse sequence [44,45]. A relaxation delay of 1.7 s was allowed between transients and the size of the matrices was 2048 x 512 complex data points. 32 scans were collected for each of the 512 experiments.

Proton-proton scalar coupling constants were extracted from 1D spectra apodized with a Gaussian function for enhancing resolution, or in case overlapping signals from the highly digitized (0.3 Hz/point) one dimensional traces of ^1^H-^1^H TOCSY spectra. 

Bruker TOPSPIN 3.0 software was used for NMR data processing.

To study the temperature dependence of amide proton resonances, one dimensional ^1^H spectra were recorded at 293, 303 and 308 K, respectively. The presence of intramolecular hydrogen bonds was assessed from the temperature gradient of amide proton resonances. NHs involved in H-bonds or pointing to the inner, sterically crowded region of the cyclic peptide are not accessible by the solvent, and therefore are exchange-protected, characterized by small temperature gradients (−1.6/−1.7 ppb/K) [46].

Complete, sequence-specific resonance assignment of proton resonances was accomplished (Table S5.) following the general strategy of Wüthrich [47], using CARA [48] (Computer Aided Resonance Assignment, Dissertation ETH Nr. 15947, by Rochus Keller, Zurich, Switzerland), a software designed for protein NMR data analysis.

Volume intensities of ROESY cross peaks were converted into structural information by distance geometry calculations using program package UNIO [49]. Automated ROESY peak picking and assignment were carried out by the ATNOS [50] and CANDID [51] algorithms. As input, the amino acid sequences, the complete sequence specific resonance assignments and the 2D ^1^H-^1^H ROESY spectral data were used. Structures were calculated by torsion angle dynamics and simulated annealing with MD software CYANA [52].

The final pools of structures were obtained in seven cycles of iterative calculation, in each cycle the experimental and calculated ROESY spectra and the obtained geometries were compared. By eliminating the wrong and/or ambiguous assignments and constraints, a good agreement was achieved for all peptides at the end of the iterative process. The quality of structures generated was characterized by several parameters, including the value of target function, the remaining number of ROE distance violations, and the average root mean square deviation (RMSD) of the bundle of conformers to the mean structures.

NMR structures were validated using the WHATIF [53] online service. The packing quality of peptides, quality of Ramachandran plot, planarity of aromatic rings and existence of anomalous bond lengths were checked. The results have confirmed that the obtained NMR ensembles contain valid peptide geometries.

#### 3.6.1. Molecular Dynamics Simulations

Refinement of the NMR structures was performed by molecular dynamics simulations without the ROE distance restraints, allowing also to characterize the dynamic behavior of peptides. The GROMACS [54] 4.5.5 software package (Groningen University, Stockholm, Sweden) with the AMBER ff03 force field parameter set was used for calculations.

The NMR-derived structural ensembles of Ac-c(CAWAC)-NH_2_ and Ac-c(CWAGC)-NH_2_ were found to be uniform, therefore starting structures for MD simulations were chosen randomly from those ensembles for these peptides. For Ac-c(CWKAC)-NH2 three well defined structural states were identified by NMR data, therefore, to reach high degree of conformational sampling, those three different starting structures, corresponding to the most populated structural states of this peptide were picked as starting structures of MD simulations. *N*-terminal acyl and *C*-terminal amide groups were added to peptide termini using Pymol 1.4.1 protein builder, and then the selected models were converted into GROMACS format. The structures were centered in a cubic, periodic simulation box (3.5 × 3.5 × 3.5 Å) of pre-equilibrated TIP3P water or DMSO [55] molecules. The systems were then energy minimized (EM) to remove atomic clashes with 1000 steps of the steepest descent method, using a 0.001 kJ/mol convergence criteria for the energy gradient. To neutralize the protonated side chain of the Lys^3^ residue in Ac-c(CWKAC)-NH_2_ a solvent molecule in the most favorable position regarding electrostatic potential was replaced by a single chloride ion. The simulation box was then subjected to EM again with the parameters mentioned above. For calculations in DMSO as solvent, the combination of conjugate gradient and steepest descent EM was necessary to reach optimum. Solvent molecules were further equilibrated by a 500 ps long MD simulation in the NVT ensemble, where heavy atoms of the solute molecule were fixed by the LINCS algorithm by applying a force constant of 1000 kJ mol^−1^ Å^2^. 

Production simulations were performed in the NPT ensemble for 100.000.000 steps with a step size of 2 fs, yielding 200-ns-long MD trajectories. To keep the bonds close to their equilibrium lengths, the LINCS constraint algorithm was used. Temperature was set at 300 K and regulated using the velocity rescaling method with a coupling time constant of 1 ps. The whole system was kept at constant pressure (1 bar), maintained by the Berendsen isotropic scaling method with a relaxation constant of 2 ps, and 5.25 × 10^−5^ bar^−1^ and 4.5 × 10^−5^ bar^−1^ isothermal compressibilities for DMSO and water, respectively. Non-bonded electrostatic interactions were calculated using the Particle–Mesh–Ewald method with all cut-off values set to 10 Å. 

Coordinates of the system were stored after every 10 ps and the first 1 ns of equilibration was excluded resulting in trajectories of 19,900 individual geometries for each peptides.

#### 3.6.2. MD Analysis

Data for structural refinement were obtained by the analysis of the MD trajectories, using the utilities of GROMACS package. Furthermore, softwares such as VMD [56], Pymol [57], Xmgrace and Microsoft Office Excel (Redmond, WA, USA) were used for visualization, statistics and generating plots.

Intramolecular hydrogen bonds were examined along the trajectories and evaluated with the g_hbond utility of GROMACS, using a 3.5 Å cut-off distance between donor and acceptor atoms and a 60° angle cut-off for donor-hydrogen-acceptor angle.

To describe the conformational properties and flexibility of the peptide backbone, secondary structure assignment was performed. The occurrence of the secondary structural elements along the trajectory was examined by the STRIDE [58] method, which analyses backbone dihedral angles and the hydrogen-bond patterns during the assignment process. 

The representative MD structures and main conformational families along the trajectories were identified by cluster analysis, fitting all atoms of the peptides, using an RMSD similarity cut-off between 0.07 nm and 0.15 nm using the gromos [59] method implemented in the g_cluster GROMACS utility.

Previously proposed intramolecular atomic distances that could play important role in the photolysis of disulfide bridges upon UV-radiation of peptides were examined in the structural ensembles.

The effect of aromatic residues was studied with peptides containing a Trp residue. The distances and their fluctuations between the sulphur atom of Cys^5^ and the C_ε_2 of the aromatic ring and the carbonyl oxygen of Trp residues have been determined along the whole trajectory. 

The conformational properties and flexibility of the side chains were described by the rotameric distributions. According to the value of χ_1_ dihedral angle extracted from the trajectories, side chains were divided into three conformational states (*gauche*+, *gauche*− and *trans*). 

### 3.7. Synthesis of Model Peptides

Linear peptides (precursors) were synthesized using Rink-Amid-MBHA resin (0.66 mmol/g) using Fmoc/*^t^*Bu protocol [33] in DMF. The coupling reaction efficiency was monitored by Kaiser test [60]. In the last step, the Fmoc protecting group was cleaved from the peptidyl-resin and the acetylation reaction of N-terminal α-amino group was performed on resin with Ac_2_O/DIEA in DMF. The final cleavage from solid support was achieved with TFA cleavage cocktail containing scavengers: TFA/TIS/Phenol/dist.water 92.5/2.5/2.5/2.5 *v*/*v*/ m%/V, 1h. The cleaved crude peptide was precipitated with ice cold diethyl ether, dissolved with water containing 5% acetic acid and lyophilised. Characteristics of the synthesized linear peptides are summarized in Table S6.

#### Cyclization of Linear Peptide Precursors through Disulfide Bond in Solution

Linear peptides were oxidized without further purification under mild alkaline conditions (0.1–0.5 mg/mL, pH 7.8–8.0 where the pH was adjusted by NH_4_HCO_3_). The cyclization reaction was monitored by analytical RP-HPLC (5 µm, 150 mm × 4.6 mm, 110 Å, Gemini-NX, Phenomenex. After lyophilisation, the crude cyclic peptides were purified by preparative RP-HPLC. All purified peptides were shown >95% homogeneity by analytical RP-HPLC and ESI-MS. Characteristics of the synthesized cyclic peptides are summarized in Table S7.

### 3.8. Fluorescence Spectroscopic Measurements

Irradiation and fluorescence measurements were performed on Jobin-Yvon Fluorolog 4 Spectrofluorometer (Kyoto, Japan) equipped with a 450-W Xenon lamp at 4 °C (by thermostat) in 1 cm × 1 cm quartz cell in argon atmosphere. The photon flux was determined by Ferrioxalate actinometry [61] at 280 and 290 nm: θ_photon_(280 nm) = 1.92.10^18^ photon/s; θ_photon_(290 nm) = 3.56/10^18^ photon/s. 

The model peptides were illuminated with 280 nm light, which was centered at 280 nm with the slit set to 16 nm band pass. The peptide solution (c = 185–220 µm in 5 mM NH_4_HCO_3_ buffer solution at pH ~ 7.5) was illuminated at 4 °C in a quartz cell (path length 1cm) with magnetic stirring (at 120 rpm) for 1 h. Before the illumination, all solutions were degassed and kept under argon atmosphere.

The evolved sulfhydryl group during the illumination of the peptide solution were quantified by fluorescence spectroscopic measurements at concentration of 35 µM peptide and in the presence of 3.5 µM CPM at 4 °C. Appropriate volume of CPM solution from DMSO stock solution was added to the irradiated peptide solution in a final concentration of 3.5 μM. The detection was based on the fluorescence increase of the peptide–CPM adduct (λ_ex_ = 387 nm, λ_emmax_ = 481 nm) The excitation was carried out at 387 nm at the 4-nm slit. The emission spectra were detected in the region of 395–680 nm. The fluorescence increase of CPM upon binding the free sulfhydryl groups was calibrated with a dilution series of linear peptide precursor Ac-CWAKC(Acm)-NH_2_.

## 4. Conclusions

Proteins, such as glycosidases, lysozymes and immunoglobulins can exhibit disulfide bridge photosensitivity [11,16]. The abundance and relative orientation of the Trp/SS motifs could have a high impact on the photosensitivity of polypeptides.

In the present study ideal small cyclic peptide models were proposed by MM in vacuum based on the following structural characteristics: i) distance between the aromatic ring and the disulfide bridge (*d*_1_ ~ 4–7 Å); ii) distance between the centroid of Trp aromatic ring and the cation center of the Arg and Lys side chain (*d*_2_/*d*_3_ ~ 5–8 Å); and iii) the angle between the Trp ring plane and the line connecting the cation and the ring center (~60–90°). In accordance with these structural criteria of the Trp-mediated photolysis, cyclic peptides (Ac-(CAWAC)-NH_2_, Ac-(CWKAC)-NH_2_ and Ac-(CWAGC)-NH_2_) were proposed as ideal models fulfilling the required criteria. Based on the pertinent W-SS distance and the presence of Lys, the following order could be proposed for the efficacy of the photolysis: Ac-(CWAGC)-NH_2_ < Ac-(CWKAC)-NH_2_ < Ac-(CAWAC)-NH_2_.

The detailed MD calculations supported by experimental NMR data also validate the expected structural characteristics of the three MM-designed Trp-containing peptides. Thus the proposed models conform the structural criteria required for efficient photolytic cleavage of the disulfide bond and so the selected peptides seemed to be suitable for studying the structural aspects of the Trp-mediated photolytic cleavage of disulfide bonds, which is the proposed mechanism of photolytic cleavage in proteins [1,2,4,13]. 

In the present study, however, we show that such events occurred only at low levels in the selected cyclic peptide models. Specifically, the results of fluorescence measurements did not support a systematic correlation between the Trp–SS distance and the amount of the detected free thiol. Instead, our results suggest the presence of other mechanisms of the photolytic cleavage of disulfide bonds in the studied peptides which was also supported by the results of LC-MS measurements, verifying the formation of oxidative photoreaction side products as well as peptide dimers and multimers.

## Figures and Tables

**Table 1:** Distances (*d*_1_, *d*_2_ as shown in Figure 1) and analytical data of the selected synthetic peptide models.

Peptide	$\frac{d_{1}}{Å}$	$\frac{d_{2}}{Å}$	Constitution	MWcalc. Monoisotopic (Measured M + H^+^)
Ac-c(CAXAC)-NH_2_				
*X*=*V*	8.26	-	C_19_H_32_N_6_O_6_S_2_	504.2 (505.3)
*X*=*W*	5.49	-	C_25_H_33_N_7_O_6_S_2_	591.2 (592.3)
Ac-c(CXAGC)-NH_2_				
*X*=*V*	6.60	-	C_18_H_30_N_6_O_6_S_2_	491.2 (492.3)
*X*=*W*	9.61	-	C_24_H_31_N_7_O_6_S_2_	577.2 (578.3)
Ac-c(CXKAC)-NH_2_				
*X*=*V*	7.76	-	C_22_H_39_N_7_O_6_S_2_	561.2 (562.3)
*X*=*W*	7.95	7.82	C_28_H_40_N_8_O_6_S_2_	648.2 (649.2)

**Table 2:** Secondary structure analysis of MD ensembles.

Ac-c(CWKAC)-NH_2_						
	Unordered	Type I β-turn	Type II β-turn	Type IV β-Turn	Inv. γ-Turn	3_10_-Helix
**H_2_O**	49%	5%	21%	18%	1%	6%
**DMSO**	58%	2%	21%	12%	-	7%
**Ac-c(CAWAC)-NH_2_**						
	**Unordered**	**Type** **I β-Turn**	**Type** **IV β-Turn**	**Type** **VIII β-Turn**	**Inv. γ-turn**	**3_10_-Helix**
**H_2_O**	60%	1%	30%	1%	8%	-
**DMSO**	74%	5%	15%	-	2%	4%
**Ac-c(CWAGC)-NH_2_**						
	**Unordered**	**Type** **i β-Turn**	**Type** **ii β-Turn**	**Type** **iv β-Turn**	**Inv.** **Γ-Turn**	**-**
**H_2_O**	45%	44%	-	11%	-	-
**DMSO**	33%	-	30%	30%	7%	-

**Table 3:** Temperature-dependent ^1^H-NMR measurements of the studied peptides, providing the chemical shift gradients of amide protons.

	NH-Chemical Shift Temperature Gradient (ppb/K) in Model Peptides				
**Ac-c(CWKAC)-NH_2_**	Cys^1^(NH)	Trp^2^(NH)	Lys^3^(NH)	Ala^4^(NH)	Cys^5^(NH)
	−5.50	−5.80	−1.70	−5.40	−5.70
**Ac-c(CAWAC)-NH_2_**	Cys^1^(NH)	Ala^2^(NH)	Trp^3^(NH)	Ala^4^(NH)	Cys^5^(NH)
	−5.90	−5.40	−1.60	−5.50	−5.50
**Ac-c(CWAGC)-NH_2_**	Cys^1^(NH)	Trp^2^(NH)	Ala^3^(NH)	Gly^4^(NH)	Cys^5^(NH)
	−5.80	−4.90	−5.60	−4.80	−5.60

**Table 4:** Analysis of Trp side-chain conformation in terms of the population of three staggered rotamers (*gauche* (+)/(−) and *trans*). Comparison of MD and NMR results.

	Gauche (+)		Gauche (−)		Trans	
**Ac-c(CWKAC)-NH_2_**	water	DMSO	water	DMSO	water	DMSO
MD	39%	25%	8%	16%	53%	59%
NMR	-	32%	-	25%	-	43%
**Ac-c(CAWAC)-NH_2_**	**Gauche (+)**		**Gauche (−)**		**Trans**	
MD	62%	78%	16%	11%	22%	12%
NMR	-	-	-	-	-	-
**Ac-c(CWAGC)-NH_2_**	**Gauche (+)**		**Gauche (−)**		**Trans**	
MD	93%	14%	4%	65%	3%	21%
NMR	-	29%	-	58%	-	12%

**Table 5:** Photodegradation results of different peptide models after 60 min illumination at 280 nm. The calculated MM distances are also shown.

Peptide 280 nm	I (480 nm)/cps ^a^	c/μM ^b^	SH% ^c^	d(SS-W)/Å	d(Lys-W)/Å
Ac-c(CWKAC)-NH_2_	519565	2.04	5.73	7.95	7.83
Ac-c(CAWAC)-NH_2_	523460	2.06	5.88	5.49	-
Ac-c(CWAGC)-NH_2_	548670	2.16	6.17	9.61	-
Ac-c(CVKAC)-NH_2_	179549	0.71	2.02	-	-

^a^ I (480 nm)/cps: fluorescence intensities of CPM-peptide adducts (average of three different measurements). ^b^ c/μM: calculated concentration of evolved sulfhydryl group based on calibration line Ac-CWKAC(Acm)-NH_2_ with CPM. ^c^ SH%: number of evolved sulfhydryl group (average of three different measurements). The 35 μM peptide concentration taken to 100%

## Supplementary Materials

The following are available online. Table S1. Characterization of the devised cyclic peptides by MM calculation, Figure S1. ^1^H-^1^H ROESY spectra of the cyclic peptides showing the sequential and medium range ROE cross peaks of NHs as well as the ROEs of the Trp residue, Table S2. NMR-ensembles and the summary of structure calculations, 4. Table S3. Analysis of MD trajectories with regard to the occurrence of hydrogen bonds. Numbers represent the population of structures in the MD ensemble possessing the specified H-bond, Table S4. Trp-fluorescence measurements of model peptides, Figure S2. CPM fluorescence I_max_ at 481 nm under various UV illumination times at 280 nm, Figure S3. Trp fluorescence emission spectra (A) λ_ex_ = 280 nm and CPM fluorescence emission spectra (B) λ_ex_ = 387 nm of Ac-c(CWKAC)-NH_2_ after irradiation for 1, 1.5, 2 and 3 h, Figure S4. CPM calibration with Ac-CWAKC(Acm)-NH_2_ peptide, Table S5. Proton (^1^H) and carbon (^13^C) chemical shifts of the studied cyclic peptides, Table S6. Analytical characteristics of linear peptides, Table S7. Analytical characteristics of cyclic peptides.
