# Supplementary material for: Photochemical and Structural Studies on Cyclic Peptide Models
Source: Molecules. 2018 Aug 30;23(9):2196. doi: 10.3390/molecules23092196 (PMC6225265; doi:10.3390/molecules23092196)
Supplement: Supplementary file 1 [file molecules-23-02196-s001.pdf]

Article

Photochemical and Structural Studies on Cyclic Peptide Models

Tamás Milán Nagy [1], Krisztina Knapp [2], Eszter Illyés [3], István Timári [1], Gitta Schlosser [4], Gabriella Csík [5], Attila Borics [6,*], Zsuzsa Majer [2,*] and Katalin E. Kövér [1,*]

[1] Department of Inorganic and Analytical Chemistry, University of Debrecen, H-4032 Debrecen, Egyetem tér 1, Hungary; tamasmilan.nagy@science.unideb.hu (T.M.N.); timari.istvan@science.unideb.hu (I.T.)
[2] Institute of Chemistry, Department of Organic Chemistry, ELTE Eötvös Loránd University, H-1518 Budapest 112. P.O. Box 32, Hungary; knkriszta@gmail.com
[3] Chemie Ltd., H-1022 Budapest, Herman Ottó út 15, Hungary; eszter@vichem.hu
[4] Department of Analytical Chemistry, Institute of Chemistry, ELTE Eötvös Loránd University, H-1518 Budapest 112, P.O. Box 32, Hungary; schlosser@caesar.elte.hu
[5] Department of Biophysics and Radiation Biology, Semmelweis University Budapest, H-1428 Budapest, P.O. Box 2, Hungary; gabriella.csik@eok.sote.hu
* Correspondence: borics.attila@brc.mta.hu (A.B.); majer@elte.hu (Z.M.); kover@science.unideb.hu (K.E.K.); Tel.: +36-52-512-900 ext. 22370 (K.E.K.)

Table of Contents

1.**Table S1.** Characterization of the devised cyclic peptides by MM calculation 3

2. **Figure S1.** ^{1}H-^{1}H ROESY spectra of the cyclic peptides showing the sequential and medium range ROE cross peaks of NHs as well as the ROEs of the Trp residue...4

3. **Table S2**. NMR-ensembles and the summary of structure calculations 5

4. **Table S3**. Analysis of MD trajectories with regard to the occurrence of hydrogen bonds. Numbers represent the population of structures in the MD ensemble possessing the specified H-bond. 6

5. **Table S4.** Trp-fluorescence measurements of model peptides..7

6. **Figure S2**. CPM fluorescence I_{max} at 481 nm under various UV illumination times at 280 nm. 8

7. **Figure S3.** Trp fluorescence emission spectra (A) λ_{ex}=280 nm and CPM fluorescence emission spectra (B) λ_{ex}= 387 nm of Ac-c(CWKAC)-NH_2 after irradiation for 1, 1.5, 2 and 3 h. 9

8. **Figure S4.** CPM calibration with Ac-CWAKC(Acm)-NH_2 peptide (λ_{ex}= 387 nm, λ_{em}= 300-600 nm). 10

9. **Table S5.** Proton (^{1}H) and carbon (^{13}C) chemical shifts of the studied cyclic peptides 11

10. **Table S6**. Analytical characteristics of linear peptides 13

11. **Table S7.** Analytical characteristics of cyclic peptides 13

1. **Table S1.** Characterization of the devised cyclic peptides by MM calculation

**Peptide**	d_1 / Å	d_2 / Å (Lys-NZ)	d_3 / Å (Arg-CZ)	**Constitution**	
**Ac-c(CAXAC)-NH$_2$**					
**X=V**	8.26	-		$C_{19}H_{32}N_6O_6S_2$	
**X=W**	5.49	-		$C_{25}H_{33}N_7O_6S_2$	**NMR**
**Ac-c(CXAGC)-NH$_2$**					
**X=V**	6.60	-		$C_{18}H_{30}N_6O_6S_2$	
**X=W**	9.61	-		$C_{28}H_{40}N_8O_6S_2$	**NMR**
**Ac-c(CXGAC)-NH$_2$**					
**X=V**	6.81			$C_{18}H_{30}N_6O_6S_2$	
**X=W**	9.68			$C_{24}H_{31}N_7O_6S_2$	
**Ac-c(CXAKC)-NH$_2$**					
**X=V**	6.57	-		$C_{22}H_{39}N_7O_6S_2$	
**X=W**	7.92	7.87		$C_{28}H_{40}N_8O_6S_2$	
**Ac-c(CXARC)-NH$_2$**					
**X=W**	9.88	-	10.45	$C_{28}H_{40}N_{10}O_6S_2$	
**Ac-c(CXKAC)-NH$_2$**					
**X=V**	7.76	-		$C_{22}H_{39}N_7O_6S_2$	
**X=W**	7.95	7.82		$C_{28}H_{40}N_8O_6S_2$	**NMR**
**Ac-c(CXRAC)-NH$_2$**					
**X=W**	9.76	-	10.80	$C_{28}H_{40}N_{10}O_6S_2$	
**Ac-c(CAXKC)-NH$_2$**					
**X=V**	8.19	-		$C_{22}H_{39}N_7O_6S_2$	
**X=W**	8.62	7.44		$C_{28}H_{40}N_8O_6S_2$	
**Ac-c(CKXAC)-NH$_2$**					
**X=V**	8.48	-		$C_{22}H_{39}N_7O_6S_2$	

2. **Figure S1.** ^1H-^1H ROESY spectra of the cyclic peptides showing the sequential and medium range ROE cross peaks of NHs as well as the ROEs of the Trp residue

a) Ac-c(CAWAC)-NH$_2$

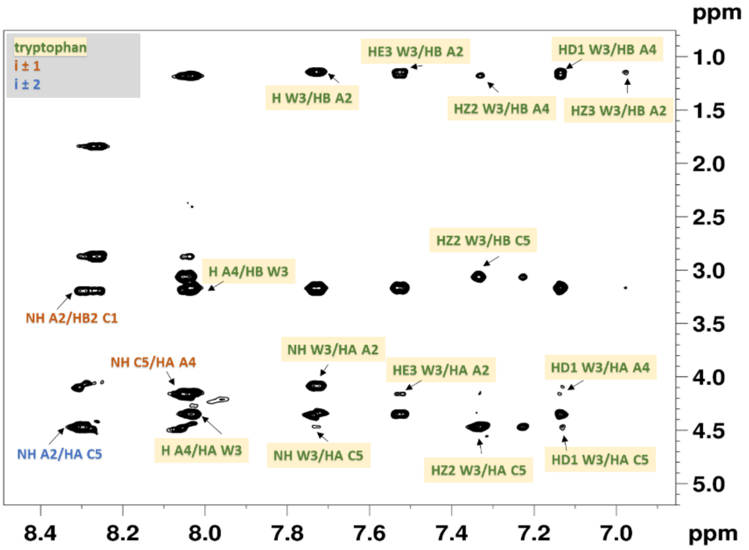

b) Ac-c(CWAGC)-NH$_2$

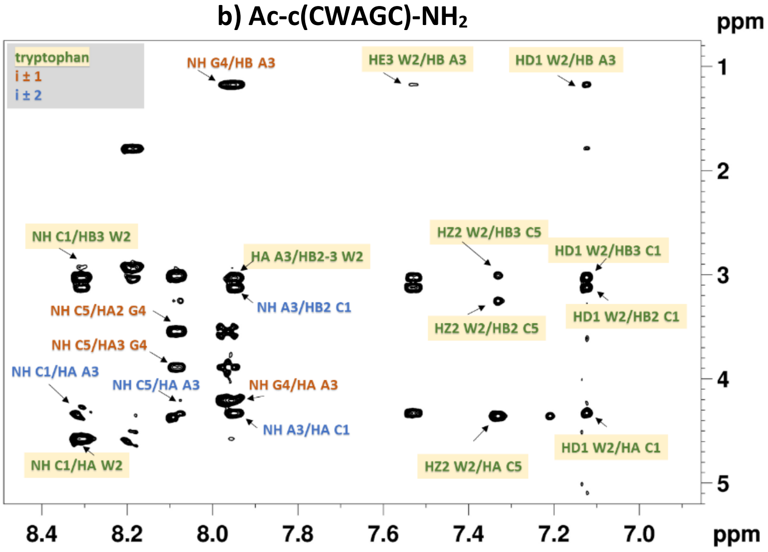

c) Ac-c(CWKAC)-NH$_2$

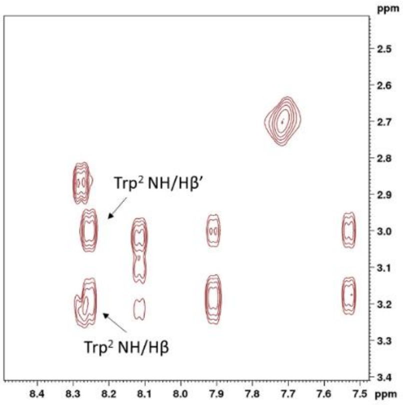

d) Ac-c(CWAGC)-NH$_2$

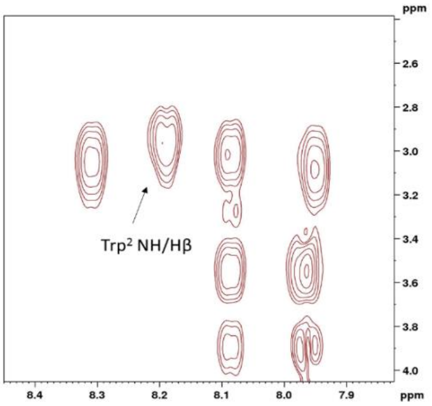

3. **Table S2**. NMR-ensembles and the summary of structure calculations

	**Ac-c(CWKAC)-NH_2**	**Ac-c(CAWAC)-NH_2**	**Ac-c(CWAGC)-NH_2**
**No. structures**	20	20	20
**RMSD to mean (backbone)**	0.74 +/- 0.21 Å	0.15 +/- 0.03 Å	0.14 +/- 0.04 Å
**NOE restaraints per residue**	8.20	11.20	8.0
**No. dihedral restraints**	20	18	18
**Secondary structures**	ß-turn type IV: 30 %<br>coil: 20 %<br>ß-turn type VIII: 5 %<br>inordered: 45 %	ß-turn type IV: 100 %	inordered: 100%
**Ramachandran statistics**	23.33 % most favoured,<br>60% additionaly allowed,<br>15 % generously allowed<br>1.67 % disallowed	21.67 % most favoured,<br>55% additionaly allowed,<br>23.33 % generously allowed<br>0 % disallowed	0 % most favoured,<br>50 % additionaly allowed,<br>50 % generously allowed<br>0 % disallowed

4. **Table S3**. Analysis of MD trajectories with regard to the occurrence of hydrogen bonds. Numbers represent the population of structures in the MD ensemble possessing the specified H-bond.

**Ac-c(CWKAC)-NH$_2$**	*Cys^1(O)-Lys^3(NH)*	*Trp^2(O)-Cys^5(NH)*	*Cys^1(O)-Ala^4(NH)*	*Lys^3(O)-Cys^5(NH)*	*NH$_2$-Cys^5(O)*	*Ac(O)-Trp^2(NH)*	*Ac(O)-Cys^1(NH)*
water	24%	-	5%	36%	100%	13%	100%
DMSO	20%	-	-	30%	100%	10%	100%
**Ac-c(CAWAC)-NH$_2$**	*Cys^1(O)-Trp^3(NH)*	*Trp^2(O)-Cys^5(NH)*	*Cys^1(O)-Ala^4(NH)*	*Trp^3(O)-Cys^5(NH)*	*NH$_2$-Cys^5(O)*	*Ac(O)-Ala^2(NH)*	*Ac(O)-Cys^1(NH)*
water	27%	-	4%	21%	100%	-	100%
DMSO	12%	-	-	43%	100%	-	100%
**Ac-c(CWAGC)-NH$_2$**	*Cys^1(O)-Ala^3(NH)*	*Trp^2(O)-Cys^5(NH)*	*Cys^1(O)-Gly^4(NH)*	*Ala^3(O)-Cys^5(NH)*	*NH$_2$-Cys^5(O)*	*Ac(O)-Trp^2(NH)*	*Ac(O)-Cys^1(NH)*
water	59%	-	10%	2%	100%	66%	100%
DMSO	29%	-	-	13%	100%	4%	100%

5. **Table S4.** Trp-fluorescence measurements of model peptides

	Peptide	Max. fluorescence intensity (cps)
1	Ac-c(CWKAC)-NH_2	82690
2	Ac-CWKAC-NH_2	108940
3	Ac-c(CAWAC)-NH_2	124660
4	Ac-CAWAC-NH_2	178975
5	Ac-c(CWAGC)-NH_2	108255
6	Ac-CWAGC-NH_2	124650

6. **Figure S2**. CPM fluorescence I_{max} at 481 nm under various UV illumination times at 280 nm.

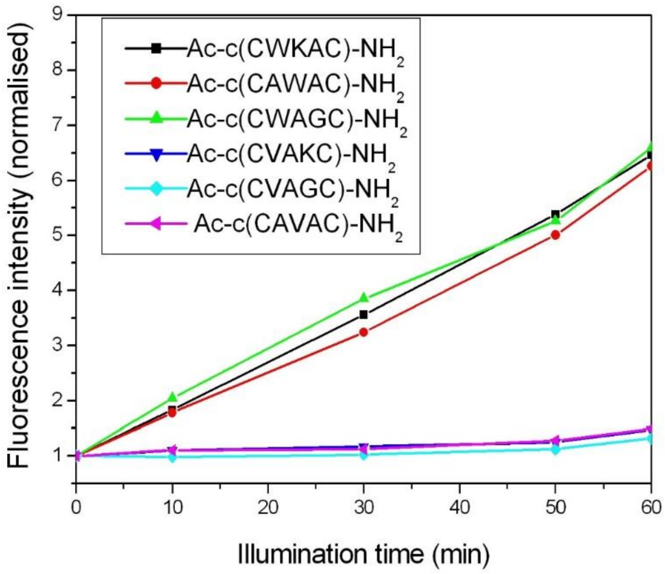

7. **Figure S3.** Trp fluorescence emission spectra (A) λ_{ex}=280 nm and CPM fluorescence emission spectra (B) λ_{ex}= 387 nm of Ac-c(CWKAC)-NH_2 after irradiation for 1, 1.5, 2 and 3 h.

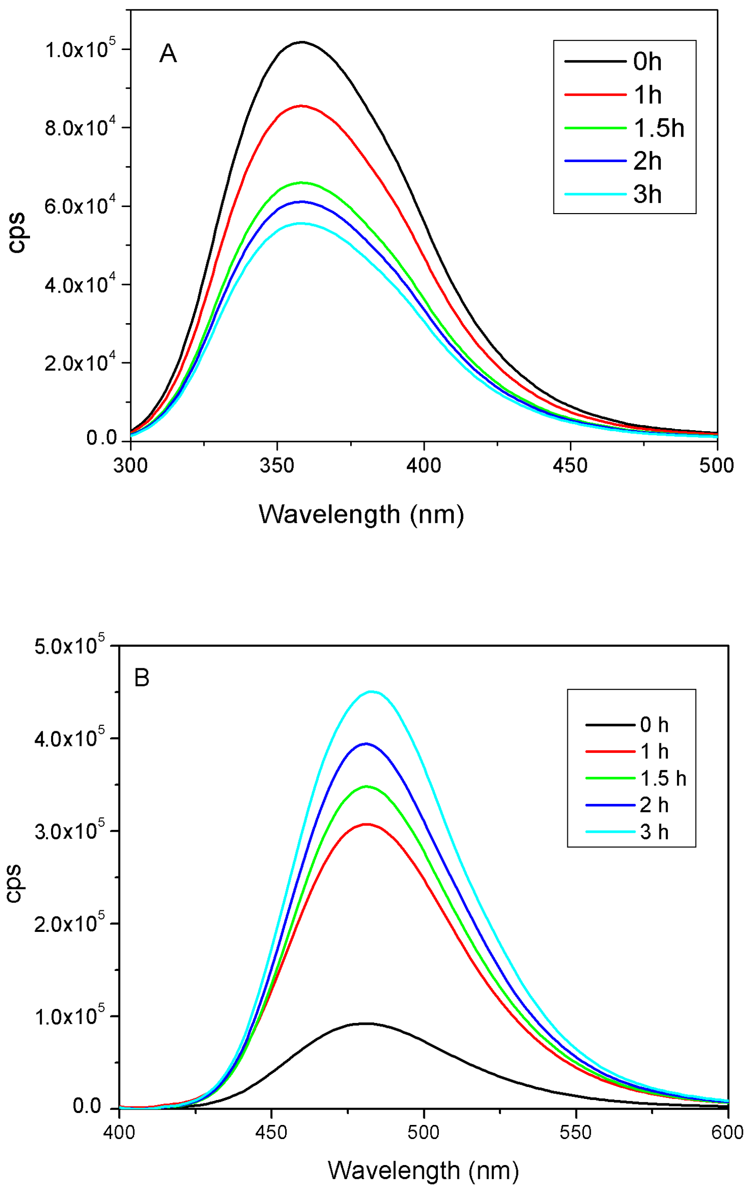

8. **Figure S4.** CPM calibration with Ac-CWAKC(Acm)-NH_2 peptide (λ_{ex}= 387 nm, λ_{em}= 300-600 nm).

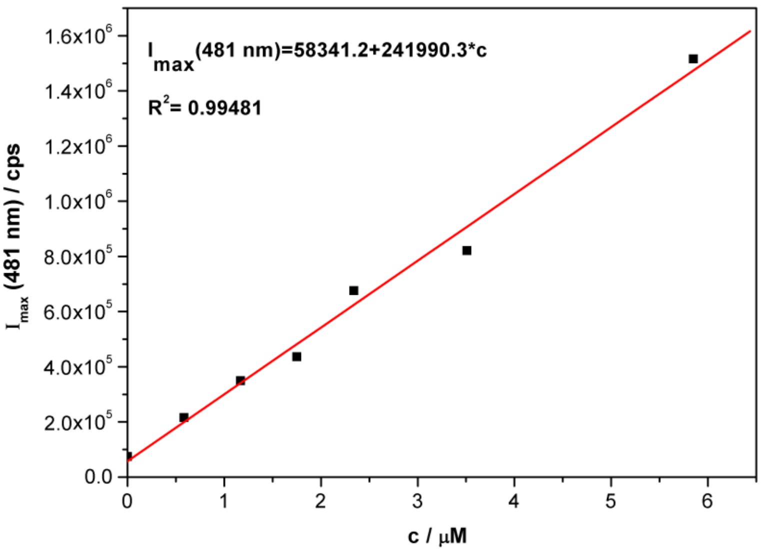

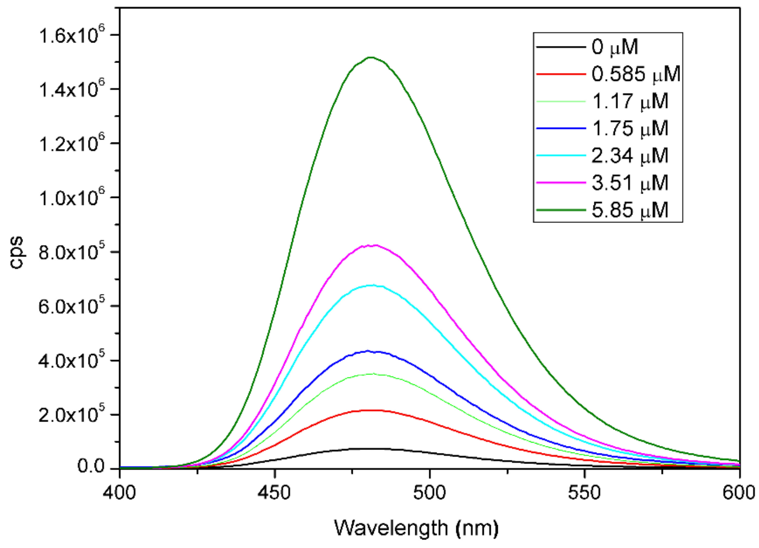

9. **Table S5.** Proton (^{1}H) and carbon (^{13}C) chemical shifts of the studied cyclic peptides

Ac-c(CWKAC)-NH₂

Cys(1) δ	Cα	=	51.84	ppm
Cys(1) δ	Cβ	=	41.97	ppm
Cys(1) δ	NH	=	8.28	ppm
Cys(1) δ	Hα	=	4.58	ppm
Cys(1) δ	Hβ	=	3.21	ppm
Cys(1) δ	Hβ'	=	2.87	ppm
Trp(2) δ	Cα	=	54.80	ppm
Trp(2) δ	Cβ	=	26.92	ppm
Trp(2) δ	CΔ1	=	123.31	ppm
Trp(2) δ	Cε3	=	117.92	ppm
Trp(2) δ	CZ	=	111.04	ppm
Trp(2) δ	CZ'	=	118.16	ppm
Trp(2) δ	CH2	=	120.80	ppm
Trp(2) δ	NH	=	8.26	ppm
Trp(2) δ	Hα	=	4.42	ppm
Trp(2) δ	Hβ	=	3.18	ppm
Trp(2) δ	Hβ'	=	3.00	ppm
Trp(2) δ	HΔ1	=	7.12	ppm
Trp(2) δ	Hε1	=	10.89	ppm
Trp(2) δ	Hε3	=	7.53	ppm
Trp(2) δ	HZ	=	7.34	ppm
Trp(2) δ	HZ'	=	6.98	ppm
Trp(2) δ	HH	=	7.07	ppm
Lys(3) δ	Cα	=	53.54	ppm
Lys(3) δ	Cβ	=	30.60	ppm
Lys(3) δ	Cγ	=	21.62	ppm
Lys(3) δ	CΔ	=	26.34	ppm
Lys(3) δ	Cε	=	38.42	ppm
Lys(3) δ	NH	=	7.91	ppm
Lys(3) δ	Hα	=	4.02	ppm
Lys(3) δ	Hβ	=	1.68	ppm
Lys(3) δ	Hβ'	=	1.59	ppm
Lys(3) δ	Hγ	=	1.14	ppm
Lys(3) δ	HΔ	=	1.46	ppm
Lys(3) δ	Hε	=	2.70	ppm
Lys(3) δ	HZ	=	7.72	ppm
Ala(4) δ	Cα	=	48.86	ppm
Ala(4) δ	Cβ	=	17.24	ppm
Ala(4) δ	NH	=	8.06	ppm
Ala(4) δ	Hα	=	4.18	ppm
Ala(4) δ	Hβ	=	1.25	ppm
Cys(5) δ	Cα	=	51.65	ppm
Cys(5) δ	Cβ	=	40.72	ppm
Cys(5) δ	NH	=	8.12	ppm
Cys(5) δ	Hα	=	4.54	ppm
Cys(5) δ	Hβ	=	3.10	ppm

Ac-c(CAWAC)-NH₂

Cys(1) δ	Cα	=	52.61	ppm
Cys(1) δ	Cβ	=	42.65	ppm
Cys(1) δ	NH	=	8.26	ppm
Cys(1) δ	Hα	=	4.49	ppm
Cys(1) δ	Hβ	=	3.20	ppm
Cys(1) δ	Hβ'	=	2.87	ppm
Ala(2) δ	Cα	=	50.19	ppm
Ala(2) δ	Cβ	=	17.69	ppm
Ala(2) δ	NH	=	8.29	ppm
Ala(2) δ	Hα	=	4.09	ppm
Ala(2) δ	Hβ	=	1.13	ppm
Trp(3) δ	Cα	=	55.32	ppm
Trp(3) δ	Cβ	=	27.40	ppm
Trp(3) δ	CΔ1	=	24.21	ppm
Trp(3) δ	Cε3	=	18.81	ppm
Trp(3) δ	CZ	=	11.78	ppm
Trp(3) δ	CZ'	=	18.75	ppm
Trp(3) δ	CH2	=	21.32	ppm
Trp(3) δ	NH	=	7.73	ppm
Trp(3) δ	Hα	=	4.35	ppm
Trp(3) δ	Hβ	=	3.17	ppm
Trp(3) δ	HΔ1	=	7.13	ppm
Trp(3) δ	Hε1	=	10.86	ppm
Trp(3) δ	HE3	=	7.53	ppm
Trp(3) δ	HZ	=	7.33	ppm
Trp(3) δ	HZ'	=	6.98	ppm
Trp(3) δ	HH2	=	7.06	ppm
Ala(4) δ	Cα	=	49.53	ppm
Ala(4) δ	Cβ	=	17.64	ppm
Ala(4) δ	NH	=	8.03	ppm
Ala(4) δ	Hα	=	4.16	ppm
Ala(4) δ	Hβ	=	1.19	ppm
Cys(5) δ	Cα	=	52.25	ppm
Cys(5) δ	Cβ	=	41.26	ppm
Cys(5) δ	NH	=	8.05	ppm
Cys(5) δ	Hα	=	4.47	ppm
Cys(5) δ	Hβ	=	3.07	ppm

Cys(5) δ Hβ' = 3.02 ppm

Ac-c(CWAGC)-NH_2

Cys(1) δ	Cα	=	55.91	ppm	Ala(3) δ	Cα	=	49.28	ppm
Cys(1) δ	Cβ	=	27.32	ppm	Ala(3) δ	Cβ	=	17.89	ppm
Cys(1) δ	NH	=	8.30	ppm	Ala(3) δ	NH	=	7.95	ppm
Cys(1) δ	Hα	=	4.33	ppm	Ala(3) δ	Hα	=	4.21	ppm
Cys(1) δ	Hβ	=	3.12	ppm	Ala(3) δ	Hβ	=	1.17	ppm
Cys(1) δ	Hβ'	=	3.04	ppm	Gly(4) δ	Cα	=	43.07	ppm
Trp(2) δ	Cα	=	52.49	ppm	Gly(4) δ	NH	=	7.96	ppm
Trp(2) δ	Cβ	=	42.27	ppm	Gly(4) δ	Hα2	=	3.55	ppm
Trp(2) δ	CΔ1	=	124.01	ppm	Gly(4) δ	Hα3	=	3.90	ppm
Trp(2) δ	Cε3	=	118.55	ppm	Cys(5) δ	Cα	=	52.42	ppm
Trp(2) δ	CZ	=	111.72	ppm	Cys(5) δ	Cβ	=	41.76	ppm
Trp(2) δ	CZ'	=	118.73	ppm	Cys(5) δ	NH	=	8.09	ppm
Trp(2) δ	CH2	=	121.34	ppm	Cys(5) δ	Hα	=	4.36	ppm
Trp(2) δ	NH	=	8.19	ppm	Cys(5) δ	Hβ	=	3.25	ppm
Trp(2) δ	Hα	=	4.58	ppm	Cys(5) δ	Hβ'	=	3.01	ppm
Trp(2) δ	Hβ	=	3.04	ppm					
Trp(2) δ	Hβ'	=	2.94	ppm					
Trp(2) δ	HΔ1	=	7.12	ppm					
Trp(2) δ	Hε1	=	10.86	ppm					
Trp(2) δ	Hε3	=	7.53	ppm					
Trp(2) δ	HZ	=	7.32	ppm					
Trp(2) δ	HZ'	=	6.97	ppm					
Trp(2) δ	HH2	=	7.05	ppm					

10. **Table S6**. Analytical characteristics of linear peptides

Linear peptide	HPLC retention time (min)	Molecular mass, calculated / measured [M+H+]	
**Ac-CAWAC-NH2**	22.0	593.7	594.6
**Ac-CWAGC-NH2**	17.5	579.7	580.6
**Ac-CWKAC-NH2**	18.9	650.8	651.7
**Ac-CVAKC-NH2**	12.0	563.7	564.3
**Ac-CWAKC(Acm)-NH2**	16.4	721.3	722.3

11. **Table S7.** Analytical characteristics of cyclic peptides

Cyclic peptide	HPLC retention time (min)	Molecular mass, calculated / measured $[M+H]^+$
Ac-c(CAWAC)-NH2	22.9	591.7 / 592.6
Ac-c(CWAGC)-NH2	16.4	577.7 / 578.6
Ac-c(CWKAC)-NH2	16.2	648.8 / 649.7
Ac-c(CVAKC)-NH2	6.0	561.7 / 562.3
